# Supplementary material for: Deregulated protein homeostasis constrains fetal hematopoietic stem cell pool expansion in Fanconi anemia
Source: Nat Commun. 2024 Feb 29;15:1852. doi: 10.1038/s41467-024-46159-1 (PMC10904799; doi:10.1038/s41467-024-46159-1)
Supplement: Supplementary file 1 — Supplementary Information [file 41467_2024_46159_MOESM1_ESM.pdf]

Supplementary Figure 1

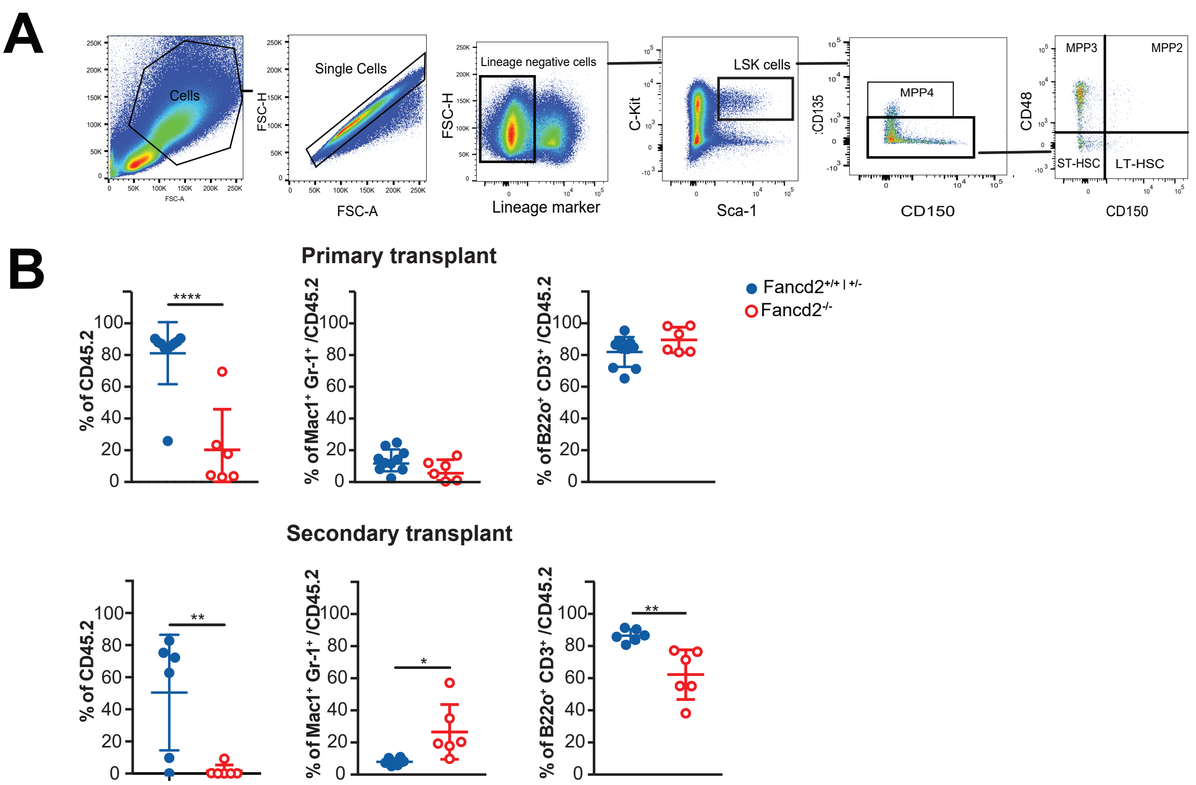

Supplementary Figure 2

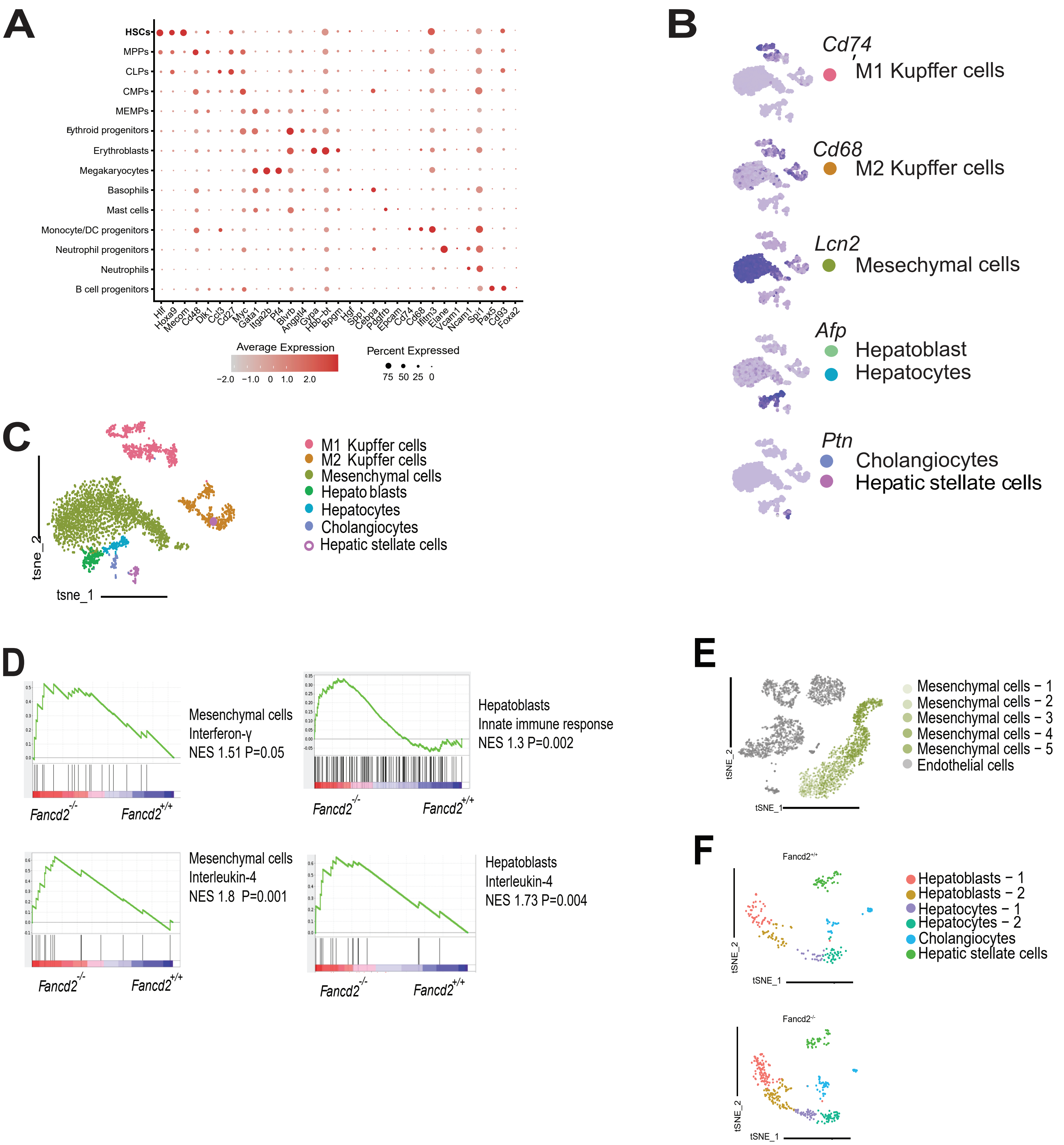

Supplementary Figure 3

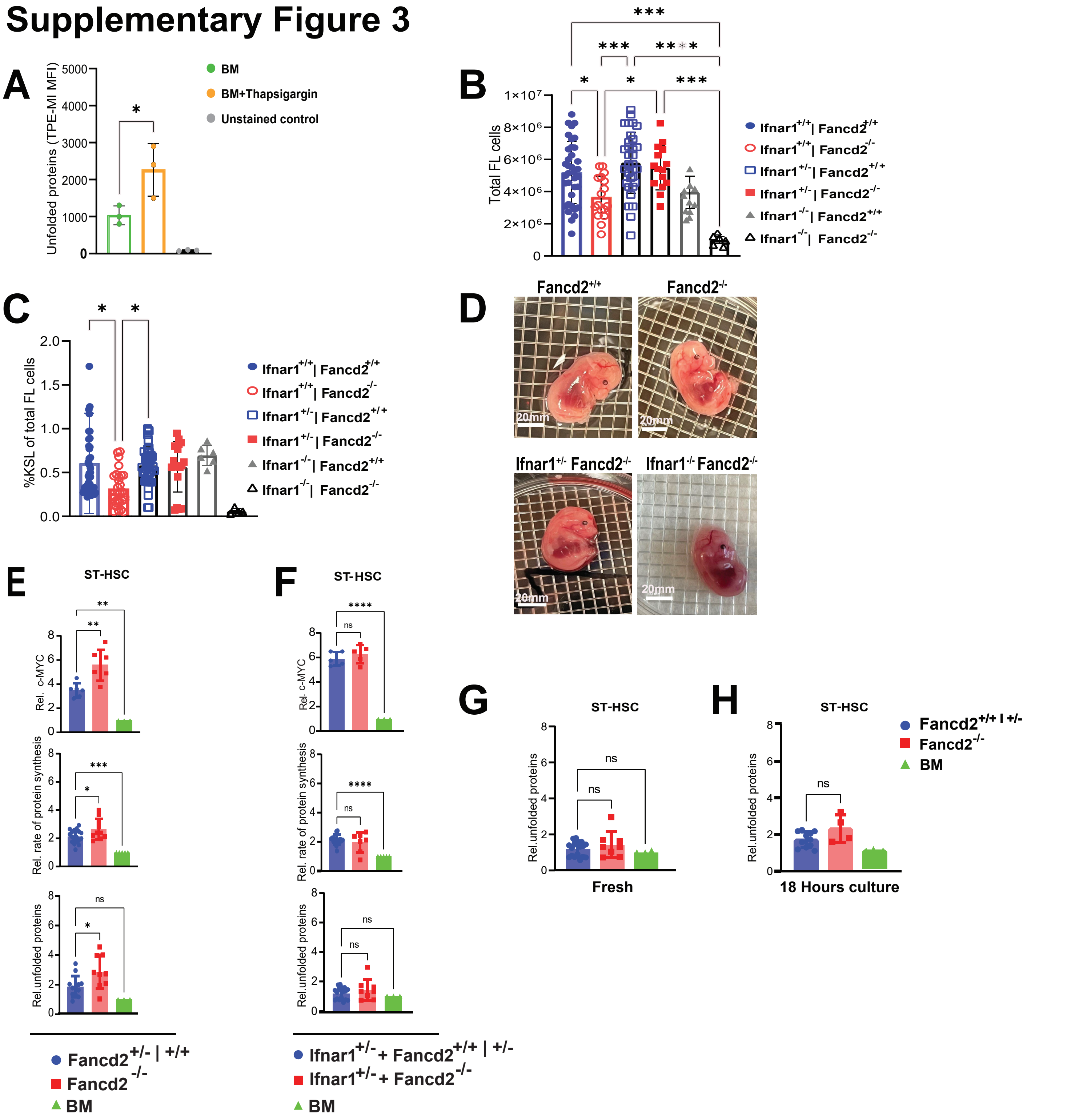

Supplemetary Figure 4

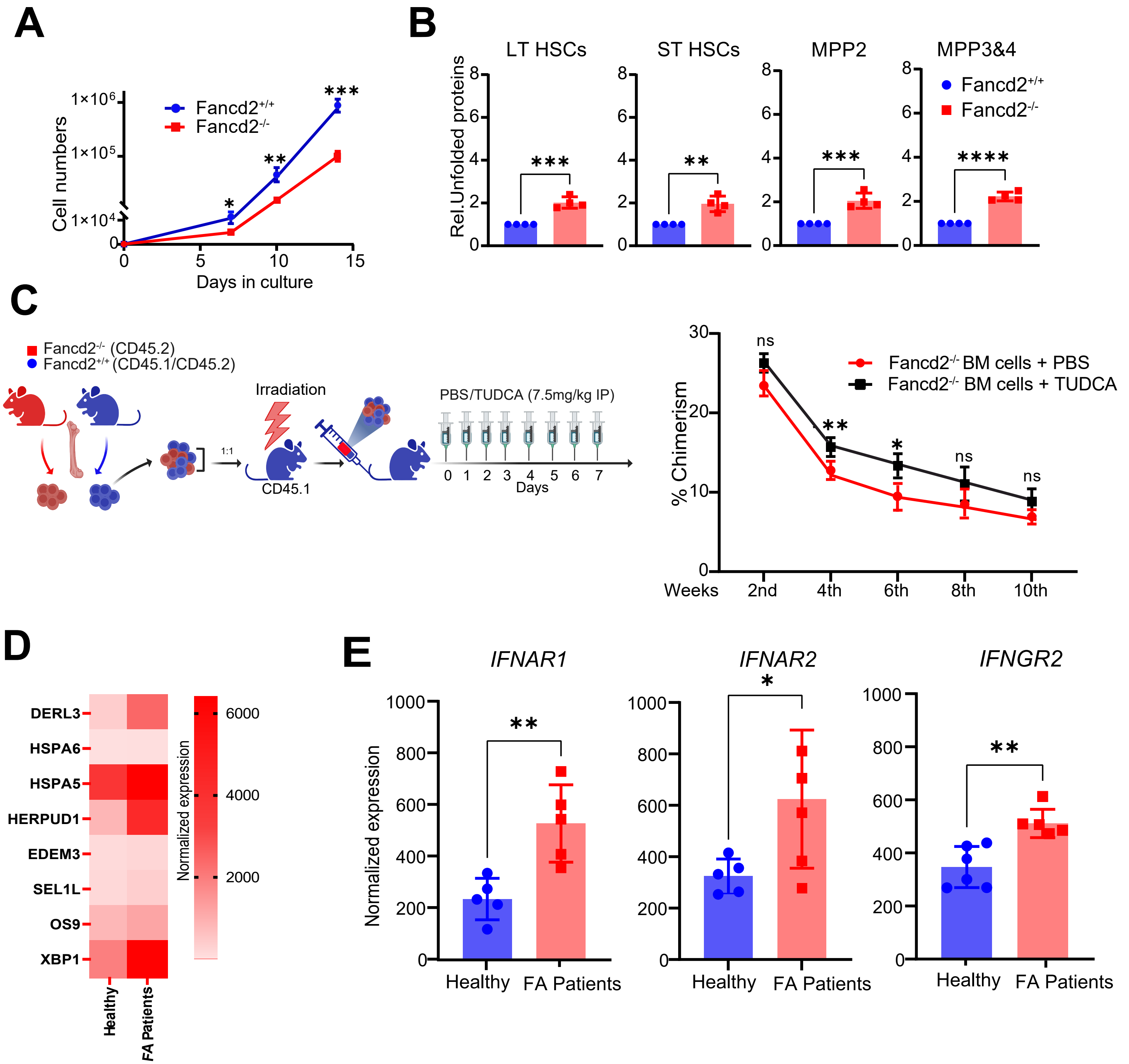

Supplementary Figure 5

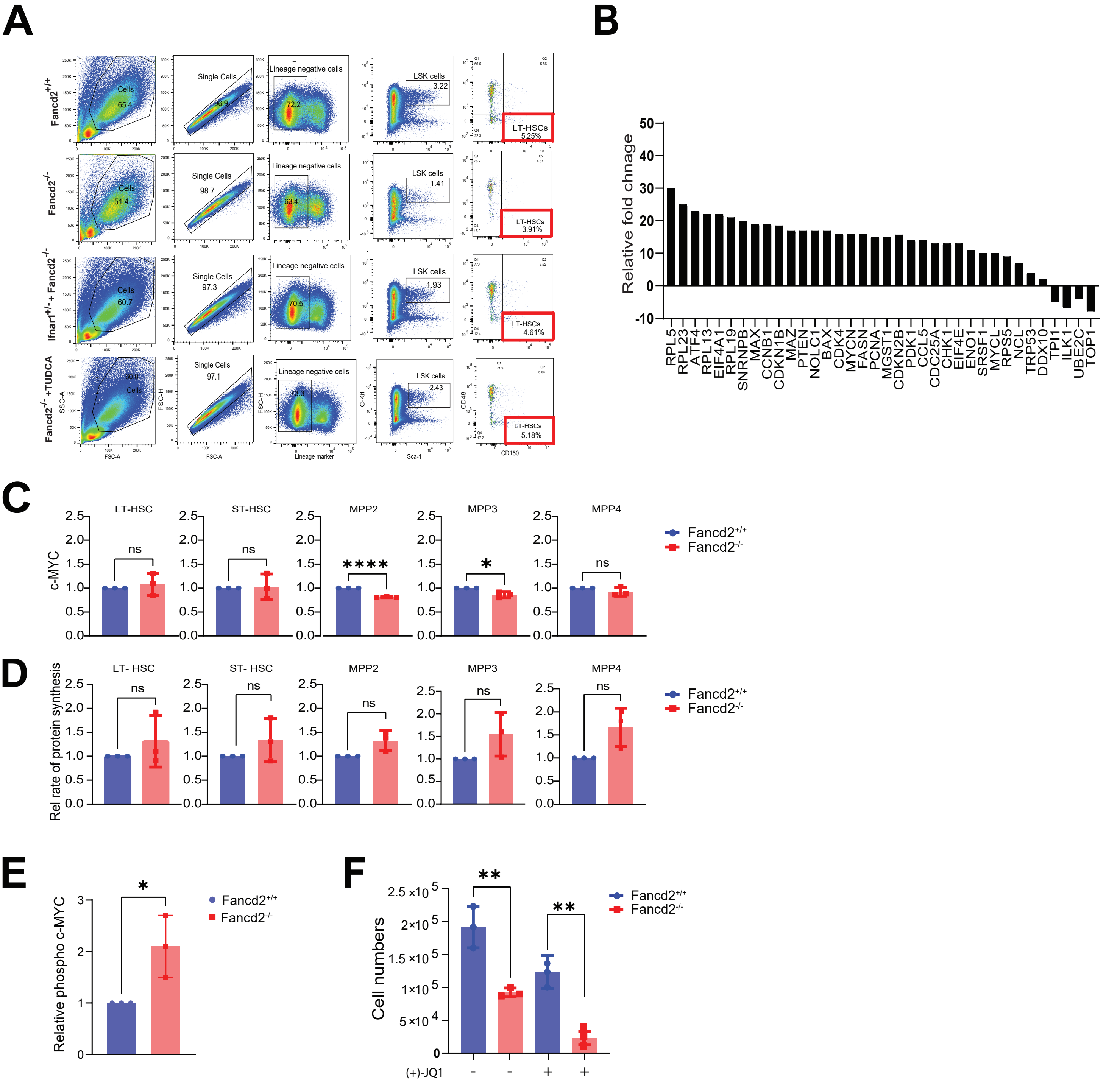

Kovuru et al., Supplementary Figure Legends

Supplementary Fig. S1: Flow cytometry gating strategy for HSPCs and transplantation analysis of E12.5 Fetal liver cells. (A) Representative flow-cytometry gating strategy for HSPC subpopulation analysis with surface staining markers. **(B)** Peripheral blood chimerism after serial transplantation using $5x10^5$ E12.5 FL Fancd2$^{+/+}$ (n=10 mice) and Fancd2$^{-/-}$ (n=6 mice). Chimerism of total CD45 (left panel), -myeloid Mac1$^+$+Gr1$^+$ (mid panel), and -lymphoid CD220+CD3 (right panel) populations 9 weeks after primary transplantation (upper panels). Chimerism 9 weeks after secondary transplantation from Fancd2$^{+/+}$ (n=6 mice) or Fancd2$^{-/-}$ (n=6 mice) donors (lower panels). Welch’s t-test for statistical analysis, *P<0.05, **P<0.01, ***P<0.001, ****P<0.0001. Source data are provided as a Source Data file.

Supplementary Fig. S2: Fetal liver niche cell composition and lineage trajectory analysis. (A) Dot plot showing the expression pattern of marker genes used for identification of hematopoietic subpopulations. **(B)** t-SNE plots illustrating the fetal liver niche cell gene expression pattern of specific marker genes. **(C**) t-SNE plot showing the global fetal liver niche for Kupffer (M1 and M2) cells, mesenchymal cells, hepatoblasts, hepatocytes, cholangiocytes, and hepatic stellate cells**. (D)** GSEA analysis shows Fancd2$^{-/-}$ mesenchymal cell enrichment for “Response to Interferon-γ” and “Interleukin-4”, and Fancd2$^{-/-}$ hepatoblast enrichment for “Innate immune response” and “Response to interleukin-4”. **(E)** Overlay of Fancd2$^{+/+}$ and Fancd2$^{-/-}$ mesenchymal and endothelial cell trajectory analyses. **(F)** Differentiation trajectory analysis of Fancd2$^{+/+}$ and Fancd2$^{-/-}$ hepatoblast. Source data have been deposited in Gene Expression Omnibus (GEO) with accession number: GSE173908. Source data are provided as a Source Data file.

Supplementary Fig. S3: Validation of TPE-MI staining in BM cells and analysis of cell count in Ifnar1$^{-/-}$ - Fancd2$^{-/-}$ crosses. (A) TPE-MI fluorescence (unfolded protein levels) increases in BM lineage-negative cells after Thapsigargin-induced endoplasmic reticulum stress conditions compared to untreated cells (n=3, t-test for statistical analysis. **(B)** Combined removal of functional Ifnar1 and Fancd2 (Ifnar1$^{-/-}$ Fancd2$^{-/-}$) alleles severely affect total FL cell counts. Ifnar1$^{+/+}$ Fancd2$^{+/+}$ (embryo numbers: n=36/8), Ifnar1$^{+/+}$ Fancd2$^{-/-}$ (n=17/8), Ifnar1$^{+/-}$ Fancd2$^{+/+}$ (n=33/), Ifnar1$^{+/-}$ Fancd2$^{-/-}$ (n=15/10), Ifnar1$^{-/-}$ Fancd2$^{+/+}$ (n=10/3) and Ifnar1$^{-/-}$ Fancd2$^{-/-}$ (n=6/3). **(C)** KSL Frequency in FL of

Ifnar1 and Fancd2 genetic combinations showing. **(D)** Representative images of E14.5 Fancd2$^{+/+}$, Fancd2$^{-/-}$, Ifnar1$^{+/-}$ Fancd2$^{-/-}$, and Ifnar1$^{-/-}$ Fancd2$^{-/-}$ embryos. **(E)** Fancd2$^{+/+ \; I+/-}$ versus Fancd2$^{-/-}$ ST-HSCs comparison of: total c-MYC protein analysis (upper panel) between Fancd2$^{+/+}$ (n=5/3) *and* Fancd2$^{-/-}$ (n=6/3), the relative rate of protein synthesis (middle panel) between Fancd2$^{+/+ \; I+/-}$ (n=5/3) *and* Fancd2$^{-/-}$ (n=6/3), and unfolded proteins (bottom panel) between the Fancd2$^{+/+ \; / \; +/-}$ (n=18/5) and Fancd2$^{-/-}$ (n=10/5). **(F)** Ifnar1$^{+/-}$ Fancd2$^{+/+I+/-}$ versus Ifnar1$^{+/-}$ Fancd2$^{-/-}$ ST-HSCs comparison of total c-MYC (upper panel) between Ifnar1$^{+/-}$ Fancd2$^{+/+}$(n=5/3) and Ifnar1$^{+/-}$ Fancd2$^{-/-}$(n=5/3), the relative rate of protein synthesis (middle panel) between Ifnar1$^{+/-}$ Fancd2$^{+/+I+/-}$ (n=17/5) and Ifnar1$^{+/-}$ Fancd2$^{-/-}$(n=7/5*)*, with unfolded proteins in the (bottom panel) comparison between the Ifnar1$^{+/-}$ Fancd2$^{+/+ \; I+/-}$ (n=17/3) vs Ifnar1$^{+/-}$ Fancd2$^{-/-}$ (n=8/3) ST-HSCs. **(G)** Analysis of unfolded proteins from Fancd2$^{+/+ \; I+/-}$ (n=19/4), and Fancd2$^{-/-}$ (n=6/4) fetal liver ST-HSCs immediately following recovery from TUDCA-injected pregnant Fancd2$^{+/-}$ mice. **(H)** ST-HSCs unfolded proteins analysis as in G, but fetal liver samples from TUDCA-injected Fancd2$^{+/-}$ mice were placed in 18 hours of ex vivo culture in the absence of TUDCA before analysis. One-way ANOVA was used for statistical analysis unless mentioned specifically. *P<0.05, **P<0.01, ***P<0.001, ****P<0.0001 and ns: non-significant. Source data are provided as a Source Data file.

Supplementary Fig. S4: Bile acid (TUDCA) supplementation improves Fancd2$^{-/-}$ cell peripheral blood chimerism, and gene expression of endoplasmic reticulum-associated degradation (ERAD) genes, unfolded protein response, and interferon receptor genes in publicly available human FA HSPCs dataset. (A) Cell proliferation kinetics comparing Fancd2$^{+/+}$ and Fancd2$^{-/-}$ BM LT-HSCs during ex vivo expansion. **(B)** Unfolded protein analysis in ex vivo expanded adult BM Fancd2$^{+/+}$ (n=3 mice), Fancd2$^{-/-}$ (n=3 mice) HSPCs subsets. t-test was used for statistical analysis. **(C)** Transient gains in peripheral blood chimerism of Fancd2$^{-/-}$ cells in recipient mice after competitive transplantation of whole bone marrow Fancd2$^{+/+}$, Fancd2$^{-/-}$ cells followed by injection of PBS (n=6 mice) or TUDCA (n=6 mice, t-test for statistical analysis). Schematic Figure was created with BioRender.com released under a Creative Commons Attribution-NonCommercial-NoDerivs 4.0 International license. **(D)** Heatmap showing the expression profile of endoplasmic reticulum-associated degradation (ERAD) and unfolded protein response genes in human FA patients HSPCs vs healthy group, data was extracted from GEO data set (GSE157591). **(E)** Increased expression of Interferon-α receptor- 1(IFNAR1), Interferon-α receptor-2 (IFNAR2), and Interferon-γ receptor-2 (IFNGR2) in human FA patients HSPCs compared to healthy HSPCs (GSE157591). Data are represented as mean ±SD. P-values of 0.01 - 0.05 were considered significant (*), 0.001 - 0.01 (**) p>0.05 were considered non-significant (ns), t-test for statistical analysis. Source data are provided as a Source Data file.

Supplementary Fig. S5: Analysis of MYC protein in adult BM cells and MYC target gene expression in fetal liver Fancd2$^{-/-}$ HSPCs. (A) Flow cytometry plots for identification of LT-HSCs from Fancd2$^{+/+}$, Fancd2$^{-/-}$, Ifnar1$^{+/-}$ Fancd2$^{-/-}$ fetal livers and Fancd2$^{-/-}$ fetal livers harvested from TUDCA-injected pregnant mice. **(B)** MYC target profiling in Fancd2$^{+/+}$ (n=12/7), Fancd2$^{-/-}$ (n=11/7) fetal liver KSL cells using "RT2 MYC profiler" PCR array. Results were normalized to Fancd2$^{+/+}$ and plotted as relative mean fold change for each target. **(C)** Relative levels of total MYC protein in age-matched adult BM LT-HSCs and HSPCs between Fancd2$^{+/+}$ (n=3 mice) and Fancd2$^{+/+}$ (n=3 mice) conditions. **(D)** Rate of protein synthesis by in vivo OPP assay in age-matched Fancd2$^{+/+}$ (n=3 mice) and Fancd2$^{-/-}$ (n=3 mice) adult BM HSPC subsets, t-test for statistical analysis in Figure panels C and D.*P<0.01, **P<0.001, ***P<0.001, ****P<0.0001. **(E)** Phosphorylated c-MYC flow cytometry analysis in day 10 ex vivo expanded (under stress conditions) adult BM HSPCs, Fancd2$^{+/+}$ (n=3 mice) and Fancd2$^{-/-}$ *(*n=3 mice), t-test for statistical analysis. **(F)** Analysis of total cell numbers in day 10 ex vivo expanded BM LT-HSCs treated with 250uM (+)-JQ1 on day 7 of culture; Fancd2$^{+/+}$ (n=3 mice) and Fancd2$^{-/-}$ (n=3 mice), (+)-JQ1 treated Fancd2$^{+/+}$ (n=3 mice) and (+)-JQ1 treated Fancd2$^{-/-}$(n=3 mice) t-test was used for statistical analysis, **P<0.001. Source data are provided as a Source Data file.

Supplementary Table 1

Antibody details with manufacturer's and catalog number details.

Antibody	Manufacturer Catalog No.	Dilution	Usage
B220 APC	Biolegend 103211	1:250	Lineage (Lin)
B220 PE	BD 12-0452-82	1:250	Lineage (Lin)
B220/CD45R FITC	Biolegend 103205	1:100	Lineage (Lin)
CD117 APC	BD 17-1171-82	1:100	c-kit
CD117 BUV395	BD Biosciences 564011	1:100	c-Kit
CD117 BV785	Biolegend 105841	1: 100	c-kit
CD117 PE	Biolegend 105808	1:100	c-kit
CD11b FITC	BD Biosciences 561691	1:100	Lineage (Lin)
CD135 BV421	Biolegend 135313	1:100	
CD150 BV711	Biolegend 115941	1:100	SLAM
CD150 PECy7	Biolegend 115914	1:100	SLAM
CD3 APC	Biolegend 100235	1:250	Lineage (Lin)
CD3 PE	BD 12-0031-82	1:250	Lineage (Lin)
CD3e FITC	Biolegend 100305	1:100	Lineage (Lin)
CD4 APC	Biolegend 100412	1:250	Lineage (lin)
CD4 FITC	Biolegend 100405	1:100	Lineage (Lin)
CD4 PE	BD Biosciences 12-0041-82	1:250	Lineage (Lin)
CD48 AF700	Biolegend 103425	1:100	
CD48 PerCp-Cy5.5	Biolegend 103422	1:100	
CD5 APC	Biolegend 100626	1:250	Lineage (Lin)
CD5 FITC	Biolegend 100605	1:100	Lineage (Lin)
CD5 PE	BD Biosciences 12-0051-82	1:250	Lineage (Lin)
c-MYC	Cell Signaling Technology 5605S	1:1000	
Gr-1 APC	Biolegend 108411	1:250	Lineage (Lin)
Gr-1 FITC	Biolegend 108405	1:100	Lineage (Lin)
Gr-1 PE	BD Biosciences 12-5931-82	1:250	Lineage (Lin)
IgG (H+L) secondary PE	ThermoFisher Scientific P-2771MP	1:100	
IgG-AF488 (anti-mouse)	Thermo Fisher A-21202	1:1000	
IgG-FITC (anti-rabbit)	SANTA CRUZ sc-2012	1:1000	
pChk1 S345	CellSignalling Technology 2348	1:50	
pMcm2 S108	Bethyl IHC-00014	1:500	
pRpa32 S4/S8	Bethyl A300-245A	1:500	
APC/ Sca-1	Biologend 122511	1:100	Sca-1
APC-Cy7/ Sca-1	Biolegend 108125	1:100	
Ter119 APC	Biolegend 116211	1:250	Lineage (Lin)
TER-119 FITC	Biolegend 116205	1:100	Lineage (Lin)
Ter119 PE	BD Biosciences 12-5921-82	1:250	Lineage (Lin)
Tgfbr1-APC	R&D FAB5871A	3:100	

Supplementary Table 2

Primers and thermocycler conditions used for genotyping and determining the sex of embryos used for single-cell RNA sequencing of murine E13.5 WT and Fancd2$^{(-/-)}$ fetal livers. Genotyping and SRY primer sequences were received from Dr. Alan D'Andrea and Dr. Sherif Abdelhamad, respectively.

Primer	Sequence (5' to 3')	Function		Thermocycler conditions
FANCD2 2F	CATGCATATAGGAACCCGAAGG	WT (forward)	genotype	95°C for 15'
FANCD2 2R	CAGGACCTTTGGAGAAGCAG	Common (reverse)		(95°C for 30", 60°C for 30", 72°C for 50") x 35
FANCD2 V76F	CTTGCAAAATGGCGTTACTTAAGC	KO (forward)		72° for 10'
Mouse SRY F	GCTGGGATGCAGGTGGAAAA	forward	sex	standard conditions
Mouse SRY R	CCCTCCGATGAGGCTGATATT	reverse		

Supplementary Table 3

Primer sequences utilized for RT-PCR experiments.

Target	Species	Sequence
IFN-α	Mouse	F- CCTGAGAGAGAAGAAACACAGCC<br>R- TCTGCTCTGACCACYTCCCAG
IL-6	Mouse	F- CCAAGAGGTGAGTGCTTCCC<br>R- CTGTTGTTCAGACTCTCTCCCT

Supplementary Table 4

Commercial assays and reagents

Assay/reagents	Source	Identification
Proteasome-Glo Chymotrypsin- like cell-based assay	Promega	Cat#G8660
MG-132	Adooq- Bioscience	Cas# 133407-82-6
O-Propargyl-Puromycin (OPP)	Medchem Source	Cat#HY-156801CS-6850
Alexa Fluor 555- Conjugated Azide	Life Technologies	Cat #A20012
Click-iT Cell Reaction Buffer Kit	Life Technologies	Cat# C10269
Tetraphenylethene maleimide(TPE-MI)	Dr. Yuning Hong	Custom- Synthesized
Proteostat-Aggresome detection kit	Enzo	Cat#ENZ-51035-K100
RNeasy plus Micro kit	Qiagen	Cat#74034
2-Mercapto ethanol	Sigma	Cat#M3148
Tauroursodeoxycholic acid (TUDCA)	Sigma-Aldrich Inc	Cat#35807-85-3
(+)-JQ1	Med Chem Express USA	HY-13030
SYBR^{TM} Green master mix	Thermo fisher-SCIENTIFIC	Cat#A25742
RT^2 Profiler PCR Array	QIAGEN	Cat# 330231 PAMM-177ZA
KAPA Mouse Genotyping Kit	Roche	Cat# KK7352
Hot Start Taq Master Mix Kit	Qiagen	Cat# 203443
collagenase I	StemCell Technologies	Cat# 07416
RBC lysis buffer	Invitrogen	Cat# 00-4300-54
Propidium Iodide (PI) solution	Miltenyi Biotec	Cat# 130-093-233
KAPA Library Quantification Kit	Roche	Cat# 07960336001
Agilent Tape Station - High Sensitivity D1000 Screen Tape	Agilent Tape Station	Cat# 5067-5584

Agilent Tape Station Reagents	Agilent Tape Station	Cat# 5067-5585
