# Supplementary material for: Deregulated protein homeostasis constrains fetal hematopoietic stem cell pool expansion in Fanconi anemia
Source: Nat Commun. 2024 Feb 29;15:1852. doi: 10.1038/s41467-024-46159-1 (PMC10904799; doi:10.1038/s41467-024-46159-1)
Supplement: Supplementary file 3 — Reporting Summary [file 41467_2024_46159_MOESM3_ESM.pdf]

nature portfolio

Corresponding author(s): Peter Kurre

Last updated by author(s): 1/17/2024

Reporting Summary

Nature Portfolio wishes to improve the reproducibility of the work that we publish. This form provides structure for consistency and transparency in reporting. For further information on Nature Portfolio policies, see our Editorial Policies and the Editorial Policy Checklist.

Statistics

For all statistical analyses, confirm that the following items are present in the figure legend, table legend, main text, or Methods section.

n/a	Confirmed	
☐	☒	The exact sample size (*n*) for each experimental group/condition, given as a discrete number and unit of measurement
☐	☒	A statement on whether measurements were taken from distinct samples or whether the same sample was measured repeatedly
☐	☒	The statistical test(s) used AND whether they are one- or two-sided<br>*Only common tests should be described solely by name; describe more complex techniques in the Methods section.*
☐	☒	A description of all covariates tested
☒	☐	A description of any assumptions or corrections, such as tests of normality and adjustment for multiple comparisons
☐	☒	A full description of the statistical parameters including central tendency (e.g. means) or other basic estimates (e.g. regression coefficient) AND variation (e.g. standard deviation) or associated estimates of uncertainty (e.g. confidence intervals)
☐	☒	For null hypothesis testing, the test statistic (e.g. *F*, *t*, *r*) with confidence intervals, effect sizes, degrees of freedom and *P* value noted<br>*Give P values as exact values whenever suitable.*
☒	☐	For Bayesian analysis, information on the choice of priors and Markov chain Monte Carlo settings
☒	☐	For hierarchical and complex designs, identification of the appropriate level for tests and full reporting of outcomes
☒	☐	Estimates of effect sizes (e.g. Cohen's *d*, Pearson's *r*), indicating how they were calculated

Our web collection on statistics for biologists contains articles on many of the points above.

Software and code

Policy information about availability of computer code

Data collection	NO software code was generated
Data analysis	Flowcytometyry samples were analyzed by FlowJo (10.6.1) software. Results were plotted as OPP median fluorescence intensity (OPP-MFI) using the GraphPad Prism 7.0 software.

For manuscripts utilizing custom algorithms or software that are central to the research but not yet described in published literature, software must be made available to editors and reviewers. We strongly encourage code deposition in a community repository (e.g. GitHub). See the Nature Portfolio guidelines for submitting code & software for further information.

Data

Policy information about availability of data

All manuscripts must include a data availability statement. This statement should provide the following information, where applicable:

- Accession codes, unique identifiers, or web links for publicly available datasets
- A description of any restrictions on data availability
- For clinical datasets or third party data, please ensure that the statement adheres to our policy

All the data points generated in this study are provided as source file and Single-cell RNA seq data have been deposited in Gene Expression Omnibus (GEO) with accession number: GSE173908. This paper does not contain original code.

Research involving human participants, their data, or biological material

Policy information about studies with human participants or human data. See also policy information about sex, gender (identity/presentation), and sexual orientation and race, ethnicity and racism.

Reporting on sex and gender	The manuscript contains no primary human data.
Reporting on race, ethnicity, or other socially relevant groupings	n/a
Population characteristics	n/a
Recruitment	n/a
Ethics oversight	n/a

Note that full information on the approval of the study protocol must also be provided in the manuscript.

Field-specific reporting

Please select the one below that is the best fit for your research. If you are not sure, read the appropriate sections before making your selection.

☒ Life sciences ☐ Behavioural & social sciences ☐ Ecological, evolutionary & environmental sciences

For a reference copy of the document with all sections, see nature.com/documents/nr-reporting-summary-flat.pdf

Life sciences study design

All studies must disclose on these points even when the disclosure is negative.

Sample size	We did not pre determine the sample size a priori. To ensure the robustness and reproducibility of our results, we make sure minimum 5 fetal livers per genotype conditions (Fancd2+/+ (WT) and Fancd2-/-(KO) harvested from a minimum of three independent pregnancies were considered for each experiment. For all the adult animal experiments we make sure that minimum 3 independent mice samples were analyzed per condition. Animal numbers were specifically reported in methods and figure legends sections respectively.
Data exclusions	Samples with inadequate numbers of cells were excluded from the data analysis.
Replication	We performed each experiment for a minimum of three times with separate pregnant dam derived Fancd2+/+ and Fancd2-/- litter mate fetal liver samples and all the data were plotted as individual data points in Figures submitted.
Randomization	Randomization occurred naturally in that the hetrozygote x heterozygote breeding strategy yields a variable number of genotype offspring, and is not impacted by investigator choice.
Blinding	Investigators were blinded while processing and analysis of these samples from each embryo harvest until we genotype embryos to identify the Fancd2 genotypic status (Fancd2+/+ or Fancd2+/- or Fancd2-/-) for post experimental data analysis.

Behavioural & social sciences study design

All studies must disclose on these points even when the disclosure is negative.

Study description	*This study didnt include any Behavioural& social sciences*
Research sample	*N/A*
Sampling strategy	*N/A*
Data collection	*N/A*
Timing	*N/A*
Data exclusions	*N/A*
Non-participation	*N/A*
Randomization	*N/A*

Ecological, evolutionary & environmental sciences study design

All studies must disclose on these points even when the disclosure is negative.

Study description	*This study didn't include any Ecological, evolutionary& environmental sciences*
Research sample	*N/A*
Sampling strategy	*N/A*
Data collection	*N/A*
Timing and spatial scale	*N/A*
Data exclusions	*N/A*
Reproducibility	*N/A*
Randomization	*N/A*
Blinding	*N/A*

Did the study involve field work? ☐ Yes ☒ No

Field work, collection and transport

Field conditions	This study did not include any field work
Location	*N/A*
Access & import/export	*N/A*
Disturbance	*N/A*

Reporting for specific materials, systems and methods

We require information from authors about some types of materials, experimental systems and methods used in many studies. Here, indicate whether each material, system or method listed is relevant to your study. If you are not sure if a list item applies to your research, read the appropriate section before selecting a response.

Materials & experimental systems

n/a	Involved in the study	
☐	☒	Antibodies
☒	☐	Eukaryotic cell lines
☒	☐	Palaeontology and archaeology
☐	☒	Animals and other organisms
☒	☐	Clinical data
☒	☐	Dual use research of concern
☒	☐	Plants

Methods

n/a	Involved in the study	
☒	☐	ChIP-seq
☐	☒	Flow cytometry
☒	☐	MRI-based neuroimaging

Antibodies

Antibodies used

Anti-CDC7 (Abcam ab108332), B220-APC (Biolegend 103211), B220-PE (BD 12-0452-82), B220/CD45R-FITC (Biolegend 103205),CD117-APC(BD 17-1171-82), CD117-BUV395 (BD Biosciences 564011), CD117-BV785 (Biolegend 105841),CD117-PE (Biolegend 105808), CD11b-FITC (BD Biosciences 561691),CD135-BV421 (Biolegend 135313), CD150-BV711(Biolegend 115941), CD150-PECy7 (Biolegend 115914), CD3-APC (Biolegend 100235, CD3-PE (BD 12-0031-82),CD3e-FITC (Biolegend 100305), CD4-APC (Biolegend 100412), CD4-FITC (Biolegend 100405), CD4-PE(BD Biosciences 12-0041-82), CD48-AF700 (Biolegend 103425), CD48-PerCp-Cy5.5 (Biolegend 103422), CD5-APC (Bio legend 100626), CD5-FITC (Biolegend 100605), CD5-PE(BD Biosciences 12-0051-82) , c-MYC (Cell Signaling Technology 5605S), gH2ax-AF488 (Biolegend 613405), Gr-1-APC (Biolegend 108411), Gr-1-FITC (Biolegend 108405), Gr-1-PE (BD Biosciences 12-5931-82), IgG (H+L) secondary PE(ThermoFisher Scientific P-2771MP), IgG-AF488 (anti-mouse),

(Thermo Fisher A-21202), IgG-FITC (anti-rabbit) (SANTACRUZ sc-2012), pChk1S345 (Cell Signaling Technology 2348), pMcm2 S108(Bethyl IHC-00014), pRpa32 S4/S8(Bethyl A300-245A), pRPA70 Invitrogen (PA5-21976), APC/ Sca-1(Biologend 122511), APC-Cy7/ Sca-1 (Biolegend 108125), Ter119 APC (Biolegend 116211), TER-119 FITC (Biolegend 116205), Ter119 PE (BD Biosciences 12-5921-82).

Validation

B220-APC (BioLegend, cat #103211, 1/100 for flow) was validated by flow cytometric analysis validation information can be found on the manufacturer's website,
B220-PE (BD, cat#12-0452-821,1/100 for flow) was validated by flow cytometric analysis in mouse bone marrow cells validation information can be found on the manufacturer's website,
B220/CD45R-FITC (Biolegend, cat#103205,1/100 for flow) was validated by flowcytometry and validation information can be found on the manufacturer's website,
CD117-APC (BD, cat#17-1171-82, 1/100, for flow) was validated by flowcytometry and validation information can be found on the manufacturer's website,
CD117-BUV395 (BD Biosciences, cat#564011, 1/100 for flow) was validated by flow cytometric analysis in mouse bone marrow cells validation information can be found on the manufacturer's website,
CD117-BV785 (Biolegend, cat#105841,1/100 for flow), was validated by flow cytometric analysis in mouse bone marrow cells validation information can be found on the manufacturer's website,
CD117-PE (Biolegend, cat#105808, 1/100 for flow), was validated by flow cytometric analysis in mouse (C57BL/6) bone marrow cells validation information can be found on the manufacturer's website,
CD11b-FITC (BD Biosciences, cat# 561691, 1/100 for flow) was validated by flow cytometric analysis in mouse bone marrow cells validation information can be found on the manufacturer's website,
CD135-BV421 (Biolegend, cat# 135313,1/100 for flow) was validated by flow cytometric analysis in mouse (C57BL/6) bone marrow cells validation information can be found on the manufacturer's website,
CD150-BV711(Biolegend, cat#115941, 1/100 for flow), was validated by flow cytometric analysis in mouse (C57BL/6) splenocytes validation information can be found on the manufacturer's website,
CD150-PECy7 (Biolegend, cat#115914,1/100) was validated by flow cytometric analysis in mouse (C57BL/6) bone marrow cells validation information can be found on the manufacturer's website,
CD3-APC (Biolegend, cat#100235 1/100), was validated by flow cytometric analysis in mouse (C57BL/6) splenocytes validation information can be found on the manufacturer's website.
CD3-PE (BD 12-0031-82, for flow 1/100) was validated by flow cytometric analysis in mouse (C57BL/6) splenocytes validation information can be found on the manufacturer's website,
CD3e-FITC (Biolegend 100305, for flow 1/100) was validated by flow cytometric analysis in mouse (C57BL/6) splenocytes validation information can be found on the manufacturer's website,
CD4-APC (Biolegend 100412, for flow 1/100), was validated by flow cytometric analysis in mouse (C57BL/6) splenocytes validation information can be found on the manufacturer's website,
CD4-FITC (Biolegend 100405, 1/100 for flow), was validated by flow cytometric analysis in mouse (C57BL/6) splenocytes validation information can be found on the manufacturer's website,
CD4-PE (BD Biosciences 12-0041-82, 1/100 for flow), was validated by flow cytometric analysis in mouse (C57BL/6) splenocytes validation information can be found on the manufacturer's website,
CD48-AF700 (Biolegend 103425,1/100 for flow), was validated by flow cytometric analysis in mouse (C57BL/6) splenocytes validation information can be found on the manufacturer's website,
CD48-PerCp-Cy5.5 (Biolegend 103422, 1/100 for flow), was validated by flow cytometric analysis in mouse (C57BL/6) splenocytes validation information can be found on the manufacturer's website,
CD5-APC (Bio legend 100626,1/250), was validated by flow cytometric analysis in mouse (C57BL/6) splenocytes validation information can be found on the manufacturer's website,
CD5-FITC (Biolegend 100605,1/100), was validated by flow cytometric analysis in mouse (C57BL/6) splenocytes validation information can be found on the manufacturer's website,
CD5-PE (BD Biosciences 12-0051-82,1/250), was validated by flow cytometric analysis in mouse (BALB/c) splenocytes validation information can be found on the manufacturer's website,
c-MYC (Cell Signaling Technology 5605S,1/1000 for flow), was validated by western blot HEK293 cells and Immunofluorescence in HeLa cells, validation information can be found on the manufacturer's website,
Gr-1-APC (Biolegend 108411,1/250 flow), was validated by flow cytometric analysis in mouse (C57BL/6) bone marrow cells validation information can be found on the manufacturer's website,
Gr-1-FITC (Biolegend 108405, 1/100 for flow), was validated by flow cytometric analysis in mouse (C57BL/6) bone marrow cells validation information can be found on the manufacturer's website,
Gr-1-PE (BD Biosciences 12-5931-82,1/250 for flow) was validated by flow cytometric analysis in mouse (BALB/c) bone marrow cells validation information can be found on the manufacturer's website.
IgG (H+L) secondary PE(ThermoFisher Scientific P-2771MP, 1/100 for flow), was validated by flow cytometric analysis of bone marrow cells, dendritic cells, and splenocytes, validation information can be found on the manufacturer's website,
IgG-AF488 (anti-mouse-Thermo Fisher A-21202, 1/1000 for IF), was validated by immunofluorescence analysis of cortical neural cells validation information can be found on the manufacturer's website,
(IgG-FITC (anti-rabbit) (SANTACRUZ sc-2012,1/1000 for IF) was validated by Immunofluorescence staining of formalin-fixed A-431 cells validation information can be found on the manufacturer's website,
pChk1S345 (Cell Signaling Technology 2348, 1/50 for IF) was validated by Confocal immunofluorescent analysis of C2C12 cells, validation information can be found on the manufacturer's website,
pMcm2 S108(Bethyl IHC-00014) was validated by immunohistochemistry in mouse squamous cell carcinoma, validation information can be found on the manufacturer's website,
pRpa32 S4/S8(Bethyl A300-245A,1/1000 for immuno histochemistry) validated in mouse gut tissue, validation information can be found on the manufacturer's website,
APC/ Sca-1(Biologend 122511, 1/100 for flow), was validated by flow cytometric analysis in mouse (C57BL/6) splenocytes validation information can be found on the manufacturer's website,
APC-Cy7/ Sca-1 (Biolegend 108125, 1/100 for flow), was validated by flow cytometric analysis in mouse (C57BL/6) splenocytes validation information can be found on the manufacturer's website,
Ter119 APC (Biolegend 116211, 1/100 for flow), was validated by flow cytometric analysis in mouse (C57BL/6) bone marrow cells validation information can be found on the manufacturer's website,
TER-119 FITC (Biolegend 116205,1/100 for flow) was validated by flow cytometric analysis in mouse (C57BL/6) bone marrow cells

validation information can be found on the manufacturer's website,
Ter119 PE (BD Biosciences 12-5921-82, for flow), was validated by flow cytometric analysis in mouse (C57BL/6) bone marrow cells validation information can be found on the manufacturer's website,

Eukaryotic cell lines

Policy information about cell lines and Sex and Gender in Research

Cell line source(s)	*This study didn't include any cell line data, we have used mouse embryonic fibroblasts (MEFs) ddetails are placed in methods sections.*
Authentication	*N/A*
Mycoplasma contamination	*These MEFs were not tested for cytoplasma contamination*
Commonly misidentified lines (See ICLAC register)	*We didnt use any cells lines in this study, so no misidentified cell lines*

Palaeontology and Archaeology

Specimen provenance	*This study didnt include any palaentology and Archaeology studies*
Specimen deposition	*N/A*
Dating methods	*N/A*

☐ Tick this box to confirm that the raw and calibrated dates are available in the paper or in Supplementary Information.

Ethics oversight	*N/A*

Note that full information on the approval of the study protocol must also be provided in the manuscript.

Animals and other research organisms

Policy information about studies involving animals; ARRIVE guidelines recommended for reporting animal research, and Sex and Gender in Research

Laboratory animals	Mice were housed in a specific pathogen-free (SPF)-grade environment with temperature ranging from 23-27°C, humidity of 30-45%. All mice were maintained under a 12-hour light/dark cycle and had free access to food and water. All experiments were performed in accordance with the Health Guide for the Care and Use of Laboratory Animals and were approved by the Biological Research Ethics Committee. we did timed pregnancies with 7 -8 week old Fancd2+/- male and female mice (C57BL/6 strain, CD45.2 isotype) to generate litter-met Fancd2+/+ and Fancd2-/- embryos, and for transplantation studies we used 7 week old B6.SJL-Ptprca Pepcb/BoyJ (CD45.1 isotype) mice as a recipients. For Studies involving IFNAR1 KO Animals (CD45.2 ,C57BL/6) were purchased from Jackson Laboratory (colony Stock No. 028288) and 7 -8 week old mice were utilized for timed pregnancies. Mice were housed in a specific pathogen-free (SPF)-grade controlled environment. ALPHA-dri was used as the cage bedding material with the room temperature ranging from 23-27°C, and humidity of 30-45%. All mice were maintained under a 12-hour light/dark cycle and had free access to sterile water and food (5015 (Lab Diet, catalog 0001328, with a metabolizable energy value of 3.59 kcal/g) in wired feeders above the floor of the cage. Animals were euthanized by exposing them to CO_2 (carbon dioxide) followed by the physical method of cervical dislocation. There was no a priori blinding of experimental cohorts at the time of analysis. The experiments described otherwise conformed to the ARRIVE guidelines for reporting animal research.
Wild animals	*This study didn't involve wild animals.*
Reporting on sex	As there is no known sex-specific FA phenotype, sex was not considered in our study design and in our approach we evaluated both male and female embryos that precludes any determination of sex bias in these observations.
Field-collected samples	*This study didn't involve samples collected from field.*
Ethics oversight	All animal experiments were approved by OHSU or CHOP Animal Care and Use Committees, respectively.

Note that full information on the approval of the study protocol must also be provided in the manuscript.

Clinical data

Policy information about clinical studies
All manuscripts should comply with the ICMJE guidelines for publication of clinical research and a completed CONSORT checklist must be included with all submissions.

Clinical trial registration	*N/A*

Study protocol	*N/A*
Data collection	*N/A*
Outcomes	*N/A*

Dual use research of concern

Policy information about dual use research of concern

Hazards

Could the accidental, deliberate or reckless misuse of agents or technologies generated in the work, or the application of information presented in the manuscript, pose a threat to:

No	Yes	
☒	☐	Public health
☒	☐	National security
☒	☐	Crops and/or livestock
☒	☐	Ecosystems
☒	☐	Any other significant area

Experiments of concern

Does the work involve any of these experiments of concern:

No	Yes	
☒	☐	Demonstrate how to render a vaccine ineffective
☒	☐	Confer resistance to therapeutically useful antibiotics or antiviral agents
☒	☐	Enhance the virulence of a pathogen or render a nonpathogen virulent
☒	☐	Increase transmissibility of a pathogen
☒	☐	Alter the host range of a pathogen
☒	☐	Enable evasion of diagnostic/detection modalities
☒	☐	Enable the weaponization of a biological agent or toxin
☒	☐	Any other potentially harmful combination of experiments and agents

Plants

Seed stocks	*This study didn't involve plants*
Novel plant genotypes	*This study didn't involve plants*
Authentication	*This study didn't involve plants*

ChIP-seq

Data deposition

☐ Confirm that both raw and final processed data have been deposited in a public database such as GEO.

☐ Confirm that you have deposited or provided access to graph files (e.g. BED files) for the called peaks.

Data access links<br>*May remain private before publication.*	*This study didn't have ChiP seq data*
Files in database submission	*This study didn't have ChiP seq data*

Genome browser session (e.g. UCSC) | This study didn't have ChiP seq data

Methodology

Replicates | This study didn't have ChiP seq data

Sequencing depth | This study didn't have ChiP seq data

Antibodies | This study didn't have ChiP seq data

Peak calling parameters | This study didn't have ChiP seq data

Data quality | his study didn't have ChiP seq data

Software | N/A

Flow Cytometry

Plots

Confirm that:

☒ The axis labels state the marker and fluorochrome used (e.g. CD4-FITC).

☒ The axis scales are clearly visible. Include numbers along axes only for bottom left plot of group (a 'group' is an analysis of identical markers).

☒ All plots are contour plots with outliers or pseudocolor plots.

☒ A numerical value for number of cells or percentage (with statistics) is provided.

Methodology

Sample preparation

Fetal livers(FL) and dam bone marrow(BM) cells were harvested Single-cell resuspended FL or BM mononuclear cells underwent red cell lysis treatment before staining with cKIT at 1:100 concentration (BioLegend 105819), SCA1 (eBioscience 25-5981-81), CD135 (BioLegend), CD48 (BioLegend 103422), and CD150 (BioLegend 115925) antibodies, as well as the lineage antibodies: B220 (BD 553090), GR1 (BioLegend 108408), CD3 (BD 555275), CD4 (BD 553653), CD5 (BD 553023), and TER119 (BD 553673) at the manufacturer's recommended concentrations. Cells were stained for 30 minutes on ice and protected from light. Blocking and washing buffer contained 2%FBS/ PBS. For viability, dead cell exclusion staining using DAPI (Thermo 62248, 1ug/ml) was included at a concentration of 1ug/ml. Flow cytometric analysis was performed using FACS Canto2 and LSR2 instruments (BD Biosciences). For intracellular staining, FL or BM mononuclear cells were stained with surface markers and fixed with 2% PFA for 15 minutes, permeabilized with 0.5% saponin, and stained with anti-p53, anti-p53S15, and anti-Cdc7. Samples were acquired with an LSR2 (Becton-Dickinson) and data were analyzed using FlowJo (10.6.1) software to quantify mean fluorescent intensity (MFI).

Instrument | Flow cytometric analysis was performed using FACS Canto2 and LSR2 instruments (BD Biosciences).

Software | Data were analyzed using FlowJo (10.6.1) software

Cell population abundance | Hematopoietic stem cells are very rare cells therefore we tried to analyze as many cells as possible by recording at least a million events total per sample

Gating strategy

In our flow cytometry analysis of pure hematopoietic stem cells (HSCs), we employed a systematic gating strategy to isolate and characterize distinct HSPC populations. To initiate the analysis, we focused on lineage-negative cells, aiming to exclude differentiated blood cells. Utilizing specific antibodies against differentiation markers, including B220, GR1, CD3, CD4, CD5, and TER119, lineage-negative cells are negative for these markers hence we identified lineage-negative cells by gating cells with negative selection for the lineage markers. Subsequently, these lineage-negative cells were further gated on c-Kit+, and Sca-1+, and positively selected for the LSK (c-Kit+ and Sca-1+) cell population. These LSK cells were further gated on FLk2(CD135), and CD150 to identify MPP4 FLk2(CD135)+ and CD150-, HSPCs(FLk2/CD135-), and these HSPCs were further gated on CD150 and CD48 for HSPCs subsets identification CD150+ and CD48- cells were gated as LT-HSCs, CD150- and CD48- cells were gated as ST-HSCs, CD150+ and CD48+ cells were gated as MPP2 and CD150- and CD48+ cells were considered as MPP3 for more clarity gating stratagy were provided as Supplementary Fig. S1A

☒ Tick this box to confirm that a figure exemplifying the gating strategy is provided in the Supplementary Information.

Magnetic resonance imaging

Experimental design

Design type | This study didnt include Magnetic resonance imaging

Design specifications | *N/A*

Behavioral performance measures | *N/A*

Acquisition

Imaging type(s) | *N/A*

Field strength | *N/A*

Sequence & imaging parameters | *N/A*

Area of acquisition | *N/A*

Diffusion MRI ☐ Used ☒ Not used

Preprocessing

Preprocessing software | *N/A*

Normalization | *N/A*

Normalization template | *N/A*

Noise and artifact removal | *N/A*

Volume censoring | *N/A*

Statistical modeling & inference

Model type and settings | *This study didnt contain any Statistical modeling &inferences*

Effect(s) tested | *N/A*

Specify type of analysis: ☐ Whole brain ☐ ROI-based ☐ Both

Statistic type for inference (See Eklund et al. 2016) | *N/A*

Correction | *N/A*

Models & analysis

n/a	Involved in the study
☒	☐ Functional and/or effective connectivity
☒	☐ Graph analysis
☒	☐ Multivariate modeling or predictive analysis

Functional and/or effective connectivity | *N/A*

Graph analysis | *N/A*

Multivariate modeling and predictive analysis | *N/A*
